# ﻿Ant-eating spiders from Xizang, China (Araneae, Zodariidae)

**DOI:** 10.3897/zookeys.1200.120528

**Published:** 2024-05-08

**Authors:** Lu-Yu Wang, Yan-Nan Mu, Feng Lu, Yong-Qiang Xu, Zhi-Sheng Zhang

**Affiliations:** 1 Key Laboratory of Eco-environments in Three Gorges Reservoir Region (Ministry of Education), School of Life Sciences, Southwest University, Chongqing 400715, China Southwest University Chongqing China; 2 Key Laboratory of Zoological Systematics and Application, College of Life Sciences, Hebei University, Baoding, Hebei 071002, China Hebei University Baoding China; 3 College of Life Sciences and Oceanography, Shenzhen University, Shenzhen 518000, China Shenzhen University Shenzhen China; 4 Tibet Plateau Institute of Biology, Lhasa 850001, Xizang Autonomous Region, China Tibet Plateau Institute of Biology Lhasa China; 5 Medog Biodiversity Observation and Research Station of Xizang Autonomous Region, Medog, China Medog Biodiversity Observation and Research Station of Xizang Autonomous Region Medog China

**Keywords:** Description, new species, morphology, taxonomy

## Abstract

Six species of the ant-eating spider of the family Zodariidae are described from Xizang, China, including five new species: *Asceuachayu***sp. nov.** (♀), *A.dawai***sp. nov.** (♂♀), *Mallinellamigu***sp. nov.** (♂), *M.mеdog***sp. nov.** (♂♀), and *M.yadong***sp. nov.** (♂♀). The female of *Cydrelalinzhiensis* (Hu, 2001) is described here for the first time. Descriptions and photographs of all the species are provided.

## ﻿Introduction

The family Zodariidae Thorell, 1881, known as ant-eating spiders, is a large spider family containing 90 genera and 1279 species worldwide, of which nine genera and 62 species have been recorded from China (WSC 2024). Among these Chinese zodariids, *Mallinella* is the largest genus, encompassing 30 known species. *Asceua* is the second largest, with 14 known species. The other genera are *Storenomorpha* with seven species, *Zodariellum* with six species, and *Cydrela*, *Euryeidon*, *Heliconilla*, *Heradion*, and *Tropizodium* with one species each.

Xizang, the second largest provincial administrative unit of China, is located in the hinterland of the Tibetan Plateau, which is known as “the roof of the world”. Xizang is also a hot spot for studying biological evolution. Knowledge of the spider diversity in Xizang is incomplete, although more than 400 species have been recorded by Hu and Li (1987a, 1987b) and Hu (2001). To date, only three zodariid species have been described from Xizang: *Cydrelalinzhiensis* (Hu, 2001) (male only, near Linzhi), *Mallinelladibangensis* (B. Biswas & K. Biswas, 2006) (female only, from southern Tibet) and *Mallinellahingstoni* (Brignoli, 1982) (female only, from “Trop de Tibet, 11,000 ft”).

During our examination of zodariid specimens from Xizang, we found five new species belonging to *Asceua* and *Mallinella*, as well as the previously unknown female of *Cydrelalinzhiensis* (Hu 2001). Here we describe these new species and the undescribed female.

## ﻿Materials and methods

All specimens are preserved in 75% ethanol and were examined, illustrated, photographed, and measured using a Leica M205A stereomicroscope equipped with a drawing tube, a Leica DFC450 Camera, and LAS software (v. 4.6). Male pedipalps and epigynes were examined and illustrated after dissection. Epigynes were cleared by immersing them in a pancreatin solution (Álvarez-Padilla and Hormiga 2007). Eye sizes were measured as the maximum dorsal diameter. Leg measurements are shown as: total length (femur, patella and tibia, metatarsus, tarsus). All measurements are in millimetres. All specimens including the holotypes examined here, are deposited in the Collection of Spiders, School of Life Sciences, Southwest University, Chongqing, China (**SWUC**).

Terminology is as follows. Abbreviations used in the text: **ALE**–anterior lateral eye; **AME**–anterior median eye; **MOA**–median ocular area; **PLE**–posterior lateral eye; **PME**–posterior median eye.

## ﻿Taxonomy

**Family Zodariidae Thorell, 1881** (拟平腹蛛科)

### ﻿Genus *Asceua* Thorell, 1887 (阿斯蛛属)

#### ﻿ Asceua chayu sp. nov.

Taxon classificationAnimaliaAraneaeZodariidae

648ED50B-85B1-5194-A0E4-CEC029371697

https://zoobank.org/325E774B-CE2D-427F-91D0-DD872F19DDE9

**Figure 1.**
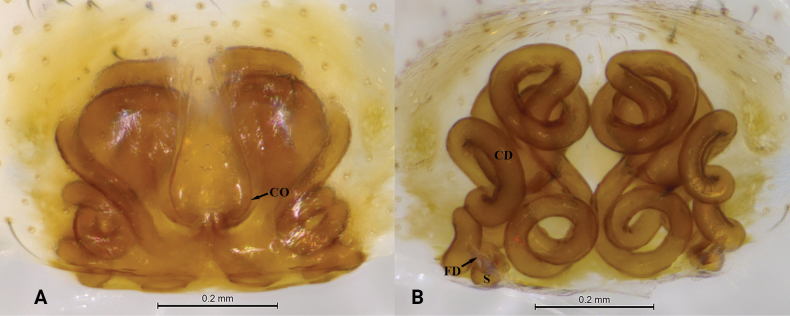
*Asceuachayu* sp. nov., holotype female **A** epigyne, ventral view **B** same, dorsal view. Abbreviations: CD = copulatory duct; CO = copulatory opening; FD = fertilization duct; S = spermatheca.

Figs 1 , 3A Chinesse name: 察隅阿斯蛛

##### Type material.

***Holotype***: China • ♀; Xizang, Chayu County, Xiachayu Town, near Xiachayu Bridge; 28°27′24″N, 97°02′40″E; elev. 1464 m; 26 June 2018; L. Wang, Z. Wu & Y. Mu leg.; SWUCT-ZOD-01-01.

##### Etymology.

The specific name is derived from the name of the county where the type locality is located; it is a noun in apposition.

##### Diagnosis.

This new species can be distinguished from all other *Asceua* species by its copulatory ducts, which are coiled more than 10 times.

##### Description.

**Female** holotype (Fig. 3A) total length 5.55. Prosoma 2.40 long, 1.62 wide; opisthosoma 2.92 long, 2.09 wide. Eye sizes and interdistances: AME 0.11, ALE 0.11, PME 0.10, PLE 0.12; AME–AME 0.08, AME–ALE 0.08, PME–PME 0.14, PME–PLE 0.21, ALE–PLE 0.06. MOA 0.42 long, anterior width 0.30, posterior width 0.35. Clypeus height 0.49. Leg measurements: I 7.44 (1.97, 2.30, 1.97, 1.20); II 6.06 (1.67, 1.80, 1.62, 0.97); III 5.96 (1.67, 1.69, 1.77, 0.83); IV 7.74 (2.01, 2.41, 2.33, 0.99). Leg formula: 4123. Carapace shiny, deep brown, lateral margins darker than median, tegument smooth, median part with an indistinct, wide V-shaped black patch in front of black fovea. Radial grooves indistinct. Opisthosoma oval, black, anterior with two pairs of wing-shaped white spots, posterior with four irregular white spots. Spinnerets brown.

***Epigyne*** (Fig. 1). Epigyne with distinct epigynal pocket, copulatory openings situated in median part of epigyne. Copulatory ducts long, visible in ventral view, coiled more than 10 times. Spermathecae small, well separated, and posteriorly situated.

##### Male.

Unknown.

##### Distribution.

Known only from the type locality, Xizang, China.

#### ﻿ Asceua dawai sp. nov.

Taxon classificationAnimaliaAraneaeZodariidae

B49FF826-98DB-5AA0-AA4E-2A71A53C7BA7

https://zoobank.org/69EA2F00-81BC-4E50-A0B4-7361B2708603

**Figure 2.**
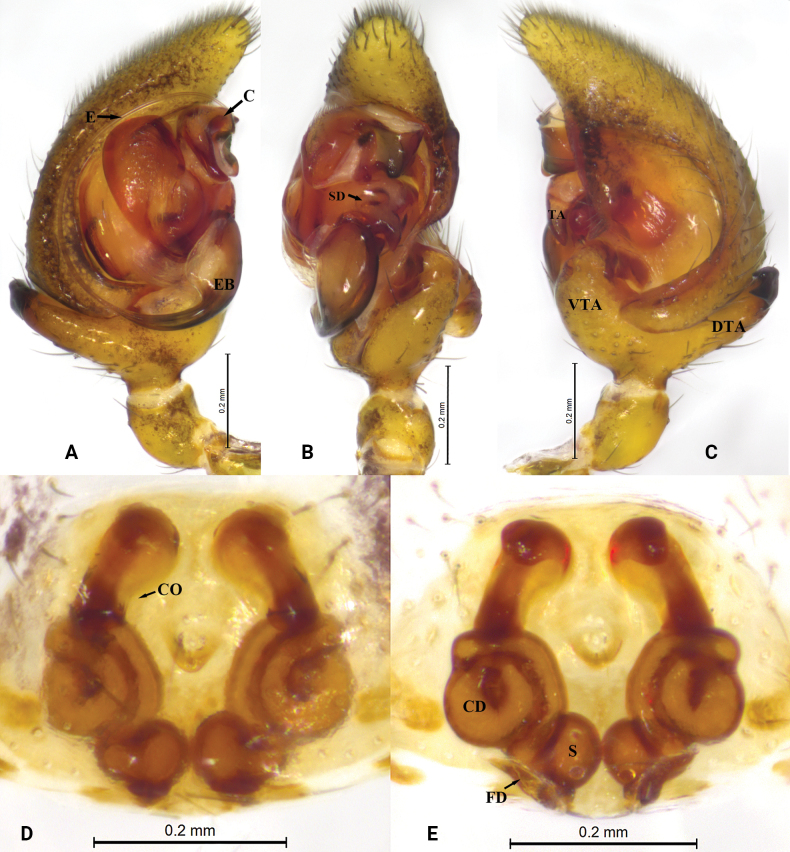
*Asceuadawai* sp. nov. **A–C** holotype male **D, E** paratype female **A** male left palp, prolateral view **B** same, ventral view **C** same, retrolateral view **D** epigyne, ventral view **E** same, dorsal view. Abbreviations: C = conductor; CD = copulatory duct; CO = copulatory opening; E = embolus; EB = embolic base; FD = fertilization duct; RTA = retrolateral tibial apophysis; S = spermatheca; SD = sperm duct; TA = tegular apophysis; VTA = ventral tibial apophysis.

**Figure 3.**
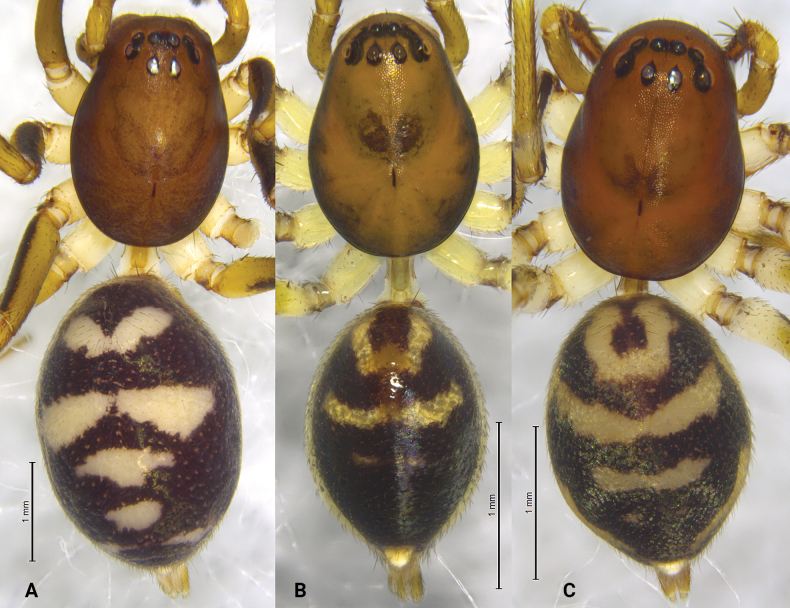
*Asceua* spp., habitus, dorsal view **A***Asceuachayu* sp. nov., female holotype **B***Asceuadawai* sp. nov., male holotype **C***Asceuadawai* sp. nov., female paratype.

Figs 2 , 3B, C Chinesse name: 达娃阿斯蛛

##### Type material.

***Holotype***: China • ♂; Xizang, Mеdog County, Medog Town, Yarang Village; 29°17′45″N, 95°16′49″E; elev. 761 m; 19 December 2023; L. Wang, F. Lu & Y. Mu leg.; SWUCT-ZOD-02-01.

***Paratypes***: China – Xizang, Mеdog County • 1 ♀; Beibeng Township; 29°14′22″N, 95°10′40″E; elev. 894 m; 28 June 2018; L. Wang, Z. Wu & Y. Mu leg.; SWUCT-ZOD-02-02 • 1 ♂; Madi Village; 29°23′42″N, 95°22′58″E; elev. 966 m; 28 June 2018; L. Wang, Z. Wu & Y. Mu leg.; SWUCT-ZOD-02-03 • 3 ♀♀; Medog Town, Yarang Village; 29°17′45″N, 95°16′49″E; elev. 761 m; 22 May 2019; L. Wang, P. Liu, T. Yuan & H. Wang leg.; SWUCT-ZOD-02-04 to SWUCT-ZOD-02-04-06 • 9 ♀♀; Medog Town, Yarang Village; 29°17′45″N, 95°16′49″E; elev. 761 m; 30 May 2022; L. Wang, B. Tan & T. Ren leg.; SWUCT-ZOD-02-07 to SWUCT-ZOD-02-15

##### Etymology.

The specific name is a patronym in honor of Mr Dawa from the Tibet Plateau Institute of Biology in Lhasa, Xizang.

##### Diagnosis.

This new species resembles *Asceuathrippalurensis* Sankaran, 2023 (Sankaran 2023: 389, figs 7A–J, 8A–J, 9A–F, 10A–F), but it differs from the latter by the short embolus that is not folded at its tip (vs long embolus folded at tip), bifurcate tip of retrolateral tibial apophysis (vs tip not bifurcate), epigynal plate with a pocket and two copulatory openings (vs without pocket and one copulatory opening), and copulatory ducts short (vs copulatory ducts long) (Fig. 2).

##### Description.

**Male** holotype (Fig. 3B) total length 3.23. Prosoma 1.44 long, 1.04 wide; Opisthosoma 1.56 long, 1.07 wide. Eye sizes and interdistances: AME 0.06, ALE 0.06, PME 0.07, PLE 0.10; AME–AME 0.06, AME–ALE 0.03, PME–PME 0.09, PME–PLE 0.10, ALE–PLE 0.04. MOA 0.27 long, anterior width 0.19, posterior width 0.25. Clypeus height 0.37. Chelicerae with 2 promarginal and 1 retromarginal tooth. Leg measurements: I 4.59 (1.21, 1.46, 1.20, 0.72); II 3.85 (1.04, 1.18, 1.03, 0.60); III 3.86 (1.10, 1.15, 1.03, 0.58); IV 4.71 (1.24, 1.54, 1.38, 0.55). Leg formula: 4132. Carapace shiny, brown, lateral margins dark brown, tegument smooth, median part with a wide V-shaped black patch in front of black fovea. Radial grooves indistinct. Opisthosoma oval, covered with short black hairs, with a shiny and lanceolate dorsal scutum. Dorsum of opisthosoma black, anterior with U-shaped white patches, followed by one transversal median band. Spinnerets brown, ringed with black.

***Palp*** (Fig. 2A–C). Tibia with strong ventral and dorsal apophyses, retrolateral tibial apophysis with curved and bifurcate tip. Tegular apophysis wide and strong, retrolaterally with coracoid extension. Embolus tapering from base to tip. Cymbium with terminal spine and with pro- and retrolateral folds not reaching tip.

**Female** (Fig. 3C) total length 3.50. Prosoma 1.69 long, 1.21 wide; opisthosoma 1.84 long, 1.35 wide. Eye sizes and interdistances: AME 0.09, ALE 0.08, PME 0.08, PLE, 0.11; AME–AME 0.05, AME–ALE 0.05, PME–PME 0.09, PME–PLE 0.12, ALE–PLE 0.06. MOA 0.29 long, anterior width 0.23, posterior width 0.28. Clypeus height 0.50. Leg measurements: I 4.52 (1.21, 1.46, 1.17, 0.68); II 3.81 (1.06, 1.17, 0.99, 0.59); III 4.01 (1.05, 1.23, 1.13, 0.60); IV 5.19 (1.31, 1.55, 1.62, 0.71). Leg formula: 4132. Opisthosoma oval, black, anterior with white U-shaped patches, followed by two transversal median bands. Spinnerets brown. Other characters same as in male, except carapace deep brown.

***Epigyne*** (Fig. 2D–E). Epigyne with a small epigynal pocket centrally, copulatory openings large, in anterior part of epigyne. Copulatory ducts short and thick, visible in ventral view. Spermathecae oval, close to each other, situated posteriorly.

##### Distribution.

Known only from the type locality, Xizang, China.

### ﻿Genus *Cydrela* Thorell, 1873 (斯逃蛛属)

#### ﻿ Cydrela linzhiensis

Taxon classificationAnimaliaAraneaeZodariidae

(Hu, 2001)

5AA257BB-EE48-5E0A-B53A-9A47C81B13D6

**Figure 4.**
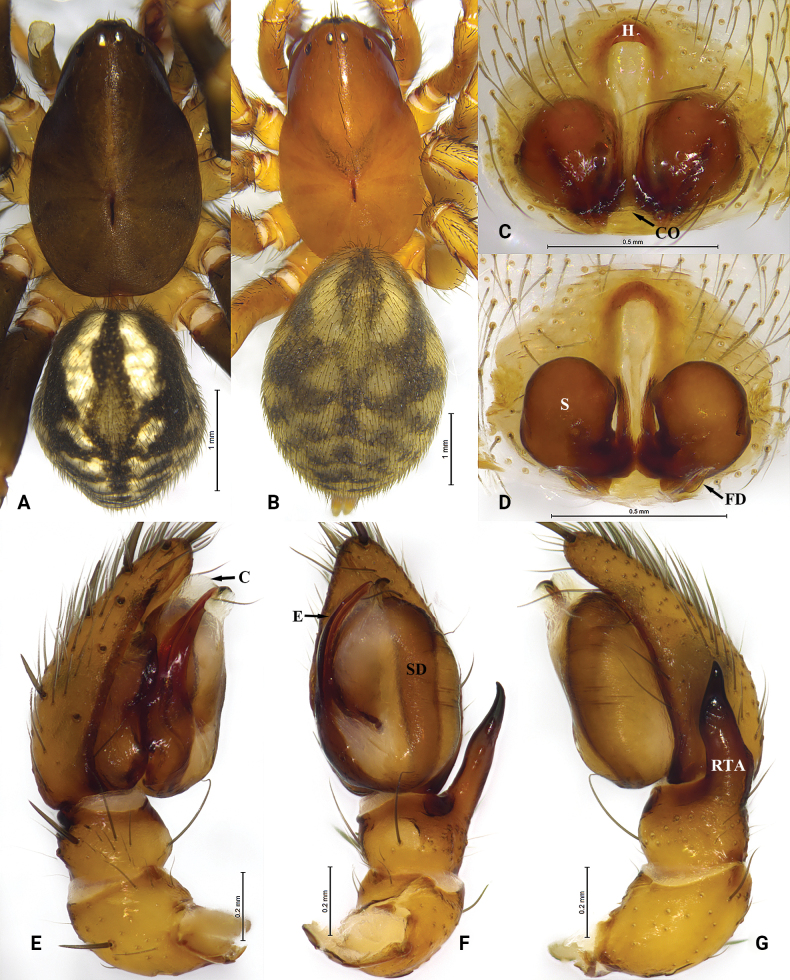
*Cydrelalinzhiensis* (Hu, 2001) **A** male, dorsal view **B** female, dorsal view **C** epigyne, ventral view **D** same, dorsal view **E** male left palp, prolateral view **F** same, ventral view **G** same, retrolateral view. Abbreviations: C = conductor; CO = copulatory opening; E = embolus; FD = fertilization duct; H = hood; RTA = retrolateral tibial apophysis; S = spermatheca; SD = sperm duct.

Fig. 4 Chinesse name: 林芝斯逃蛛

Storena linzhiensis Hu, 2001: 92, figs 14.1–6 (♂).
Cydrela linzhiensis : Dankittipakul and Jocqué 2006: 100.

##### Material examined.

China – Xizang • 1 ♀; Nyingchi City, Bowo County, Tongmai Town, near Tongmai Bridge; 30°05′41″N, 95°04′13″E; elev. 2073 m; 1 July 2018; L. Wang, Z. Wu & Y. Mu leg.; SWUC-ZCL-01 • 1 ♂; near Tongmai Bridge; L. Wang, P. Liu, T. Yuan & H. Wang leg.; SWUC-ZCL-02.

##### Diagnosis.

This species can be easily separated from other *Cydrela* species by the long retrolateral tibial apophysis of the male palp and large, globular spermathecae of the epigyne.

##### Description.

**Male** (Fig. 4A) total length 4.90. Prosoma 2.74 long, 1.72 wide; Opisthosoma 2.11 long, 1.70 wide. Eye sizes and interdistances: AME 0.06, ALE 0.09, PME 0.09, PLE 0.11; AME–AME 0.05, AME–ALE 0.04, PME–PME 0.09, PME–PLE 0.18, ALE–PLE 0.24. MOA 0.29 long, anterior width 0.17, posterior width 0.26. Clypeus height 0.48. Leg measurements: I 6.40 (1.82, 2.18, 1.36, 1.04); II 5.90 (1.64, 1.92, 1.31, 1.03); III 6.04 (1.64, 1.85, 1.73, 0.82); IV 8.31 (2.14, 2.33, 2.47, 1.37). Leg formula: 4132. Carapace brown. Fovea dark red. Radial grooves indistinct. Opisthosoma oval, black, with short hairs, dorsum with five pairs of bright spots, anteriormost one largest. Spinnerets yellow brown.

***Palp*** (Fig. 4E–G). Tibia as long as wide, protruded ventrally in lateral view, retrolateral tibial apophysis long, with wide base and triangular tip, two times longer than tibia. Cymbium with several spines on distal part. Bulb oval. Conductor membranous, posterior portion lightly sclerotized; anterior part forming a groove accommodating elongate embolus.

**Female** (Fig. 4B) total length 6.95. Prosoma 3.42 long, 2.08 wide; opisthosoma 3.66 long, 2.63 wide. Eye sizes and interdistances: AME 0.08, ALE 0.11, PME 0.10, PLE, 0.11; AME–AME 0.06, AME–ALE 0.05, PME–PME 0.11, PME–PLE 0.28, ALE–PLE 0.38. MOA 0.39 long, anterior width 0.21, posterior width 0.32. Clypeus height 0.47. Leg measurements: I 6.05 (1.81, 2.24, 1.14, 0.86); II 5.28 (1.61, 1.79, 1.05, 0.83); III 5.86 (1.70, 1.87, 1.34, 0.95); IV 8.04 (2.14, 2.51, 2.07, 1.32). Leg formula: 4132. Carapace deep yellow, opisthosoma gray, other characters same as male.

***Epigyne*** (Fig. 4C, D). Epigynal plate with small hood, wider than long. Copulatory opening conspicuous, posteriorly situated. Copulatory ducts short. Spermathecae large, globular.

##### Distribution.

China (Nyingchi, Xizang).

### ﻿Genus *Mallinella* Strand, 1906 (马利蛛属)

**The *fronto* -group**

#### ﻿ Mallinella migu sp. nov.

Taxon classificationAnimaliaAraneaeZodariidae

75E068FE-D435-5F9A-B3CF-EB8B42AECED0

https://zoobank.org/3A85835E-B332-46D6-AC02-3C2F84F2EAF2

**Figure 5.**
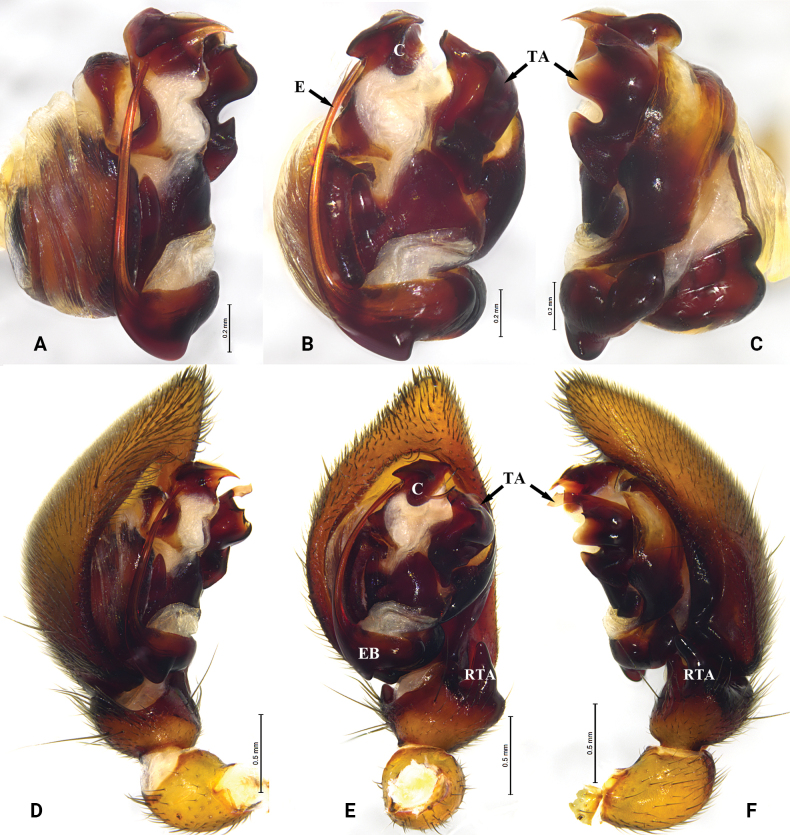
*Mallinellamigu* sp. nov. holotype male **A** male right bulb (flipped horizontally), prolateral view **B** same, ventral view **C** same, retrolateral view **D** male left palp, prolateral view **E** same, ventral view **F** same, retrolateral view. Abbreviations: C = conductor; E = embolus; EB = embolic base; RTA = retrolateral tibial apophysis; TA = tegular apophysis.

Figs 5 , 10A Chinesse name: 米古马利蛛

##### Type material.

***Holotype***: China • ♂; Xizang, Chayu County, Shangchayu Town, Migu; 28°46′40″N, 96°43′28″E; elev. 1945 m; 27 May 2019; L. Wang, P. Liu, T. Yuan & H. Wang leg.; SWUCT-ZOD-03-01.

##### Etymology.

The specific name is derived from the type locality; it is a noun in apposition.

##### Diagnosis.

This new species resembles *Mallinellamartensi* (Ono, 1983) (Ono 1983: 212, figs 1–4), but it differs from the latter by the stronger and wider tegular apophysis with a narrow, deep notch at external rim in retrolateral view (vs thin tegular apophysis with a wide notch), conductor with a nearly rectangular apophysis at prolateral base (vs with cambered apophysis), embolic base with a distinct, blunt protuberance (vs without protuberance) (Fig. 5).

##### Description.

**Male** holotype (Fig. 10A) total length 9.57. Prosoma 4.81 long, 3.46 wide; Opisthosoma 4.35 long, 3.10 wide. Eye sizes and interdistances: AME 0.31, ALE 0.24, PME 0.25, PLE 0.26; AME–AME 0.12, AME–ALE 0.19, PME–PME 0.21, PME–PLE 0.36, ALE–PLE 0.08. MOA 0.68 long, anterior width 0.63, posterior width 0.70. Clypeus height 1.26. Leg measurements: I 14.10 (3.62, 4.24, 3.53, 2.71); II 13.00 (3.40, 3.91, 3.23, 2.46); III 12.52 (3.17, 3.71, 3.55, 2.09); IV 16.09 (3.80, 4.66, 4.92, 2.71). Leg formula: 4123. Carapace and fovea black, slightly swollen. Legs yellow. Opisthosoma oval, longer than wide, dorsum with two pairs of white spots, followed by three transverse white bands. Dorsal scutum thin, reddish brown, about half as long as opisthosoma. Spinnerets pale yellow.

***Palp*** (Fig. 5). Tibia wider than long, with two apophyses, ventral tibial apophysis arc-shaped; retrolateral tibial apophysis digitiform, short, apically rounded, curved ventrally. Tegular apophysis wide, apico-prolateral process strongly curved, with thin, deep notch at external rim in retrolateral view. Embolus bifurcated at tip, lateral ramus shorter than mesal ramus, with blunt protuberance at base. Conductor sclerotized posteriorly; anterior part forming a groove accommodating embolus.

**Female.** Unknown.

##### Distribution.

Known only from the type locality, Xizang, China.

#### ﻿ Mallinella mеdog sp. nov.

Taxon classificationAnimaliaAraneaeZodariidae

0A747C0B-B4BF-57A6-B863-3CDAED88E5F0

https://zoobank.org/DA6D56B6-4AE4-44BC-8865-8A49415C428B

**Figure 6.**
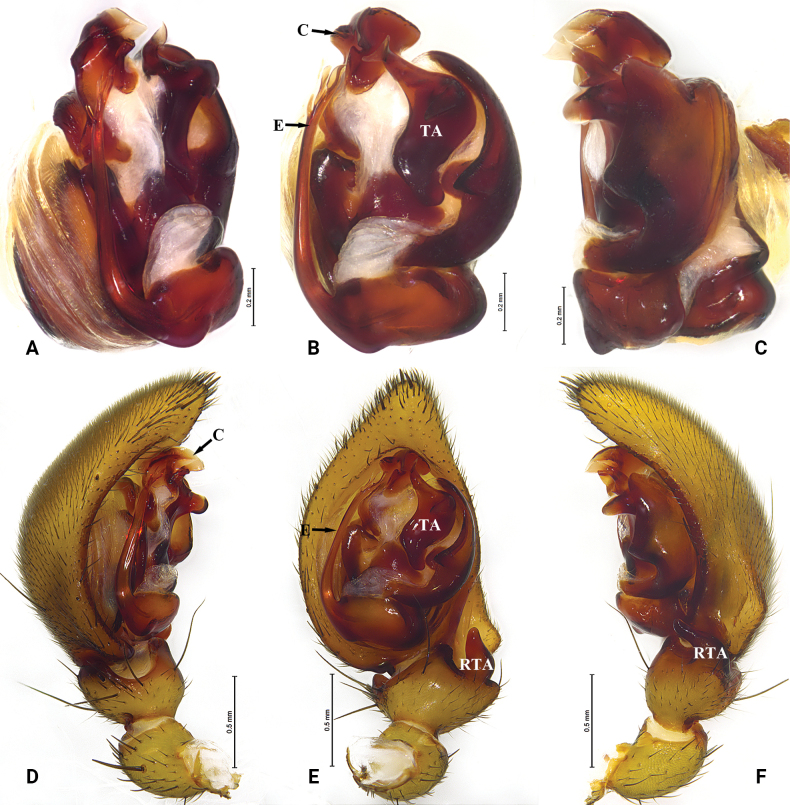
*Mallinellamеdog* sp. nov. **A–C** paratype male **D–F** holotype male **A** male left bulb, prolateral view **B** same, ventral view **C** same, retrolateral view **D** male left palp, prolateral view **E** same, ventral view **F** same, retrolateral view. Abbreviations: C = conductor; E = embolus; RTA = retrolateral tibial apophysis; TA = tegular apophysis.

**Figure 7.**
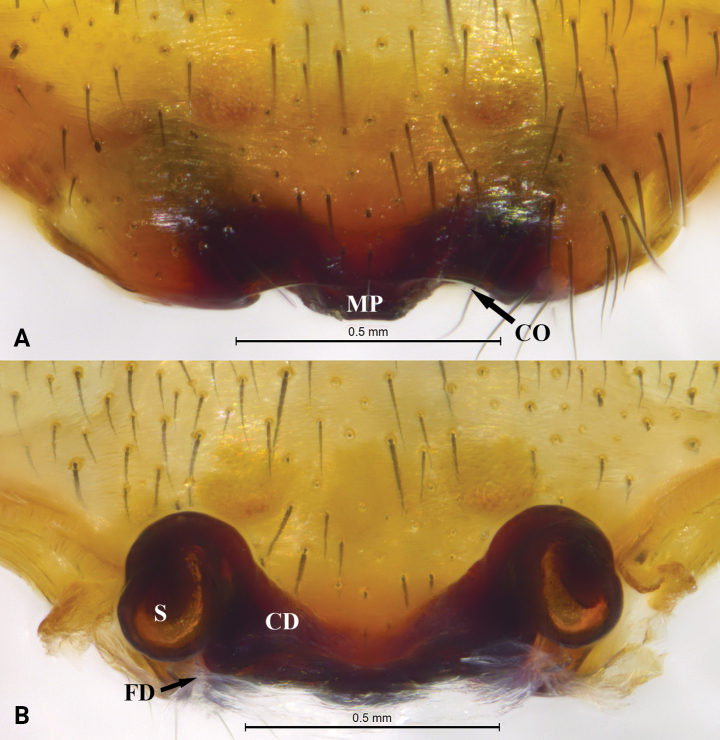
*Mallinellamеdog* sp. nov. paratype female **A** epigyne, ventral view **B** same, dorsal view. Abbreviations: CD = copulatory duct; CO = copulatory opening; FD = fertilization duct; MP = median plate; S = spermatheca.

Figs 6 , 7 , 10B, C Chinesse name: 墨脱马利蛛

##### Type material.

***Holotype***: China • ♂; Xizang, Mеdog County, near Dexing Bridge; 29°19′16″N, 95°17′39″E; elev. 724 m; 29 June 2018; L. Wang, Z. Wu & Y. Mu leg.; SWUCT-ZOD-04-01.

***Paratypes***: China – Xizang, Mеdog County • 1♂ 1♀; Mеdog County; 29°19′28″N, 95°19′37″E; elev. 1116 m; 29 June 2018; L. Wang, Z. Wu & Y. Mu leg.; SWUCT-ZOD-04-02 and SWUCT-ZOD-04-03 • 1♂ 1♀; Medog Town, Yarang Village; 29°17′45″N, 95°16′49″E; elev. 761 m; 22 May 2019; L. Wang, P. Liu, T. Yuan & H. Wang leg.; SWUCT-ZOD-04-04 and SWUCT-ZOD-04-05 • 3♂♂ 3♀♀; Medog Town, Yarang Village; 29°17′45″N, 95°16′49″E; elev. 761 m; 30 May 2022; L. Wang, B. Tan & T. Ren leg.; SWUCT-ZOD-04-06 to SWUCT-ZOD-04-11 • 3♀♀; Mеdog County; 29°19′37″N, 95°19′33″E; elev. 1049 m; 31 May 2022; L. Wang, B. Tan & T. Ren leg.; SWUCT-ZOD-04-12 to SWUCT-ZOD-04-14 • 2♂♂; Medog Town; 29°19′36″N, 95°19′16″E; elev. 1008 m; 6 July 2023; L. Wang, F. Lu & X. Chen leg.; SWUCT-ZOD-04-15 and SWUCT-ZOD-04-16 • 10♂♂ 5♀♀; Beibeng Township, Badeng Village; 29°16′28″N, 95°10′7″E; elev. 851 m; 7 July 2023; L. Wang, F. Lu & X. Chen leg.; SWUCT-ZOD-04-17 to SWUCT-ZOD-04-31 • 1♂; Guoguo Tang; 29°19′10″N, 95°16′54″E; elev. 855 m; 8 July 2023; Z. Zhang, L. Wang, F. Lu & X. Chen leg.; SWUCT-ZOD-04-32 • 6♂♂ 2♀♀; Haishishenlou observation deck; 29°20′36″N, 95°20′43″E; elev. 1297 m; 8 July 2023; Z. Zhang, L. Wang, F. Lu & X. Chen leg.; SWUCT-ZOD-04-33 to SWUCT-ZOD-04-40.

##### Etymology.

The specific name is derived from the type locality; it is a noun in apposition.

##### Diagnosis.

The male of this new species resembles *Mallinellamartensi* (Ono 1983: 212, figs 1–4) and *M.migu* sp. nov., but it differs from these two species by the anterior of tegular apophysis rostrated in ventral view (vs not rostrated), retrolateral tibial apophysis curved ventrally in retrolateral view (vs vertical, not curved) (Fig. 6). The female of this new species resembles *M.sphaerica* Jin & Zhang, 2013 (Jin and Zhang 2013: 81, figs 7, 8, 12, 13) but differs by the wider copulatory ducts (vs thin copulatory ducts) (Fig. 7).

##### Description.

**Male** holotype (Fig. 10B) total length 7.23. Prosoma 3.84 long, 2.60 wide; Opisthosoma 3.35 long, 2.28 wide. Eye sizes and interdistances: AME 0.30, ALE 0.21, PME 0.20, PLE 0.21; AME–AME 0.17, AME–ALE 0.12, PME–PME 0.16, PME–PLE 0.32, ALE–PLE 0.05. MOA 0.68 long, anterior width 0.65, posterior width 0.57. Clypeus height 0.88. Chelicerae with 1 promarginal and 3 retromarginal teeth. Leg measurements: I 13.24 (3.30, 3.88, 3.57, 2.49); II 12.12 (3.07, 3.53, 3.31, 2.21); III 11.39 (2.76, 3.29, 3.43, 1.91); IV 14.58 (3.42, 4.08, 4.65, 2.43). Leg formula: 4132. Carapace brown; fovea deep red, slightly swollen. Legs yellow. Opisthosoma oval, longer than wide, dorsum with two pairs of white spots, followed by two transverse white bands. Dorsal scutum thin, reddish brown. Spinnerets yellow.

***Palp*** (Fig. 6). Tibia with two apophyses, ventral tibial apophysis small, arc-shaped; retrolateral tibial apophysis digitiform, strongly curved ventrally. Tegular apophysis wide at middle parts, apico-prolateral process rostrate, posterior process blunt. Embolus bifurcate, lateral ramus shorter than mesal ramus. Conductor sclerotized, apex of conductor irregularly fluctuating.

**Female** (Fig. 10C) total length 10.00. Prosoma 4.65 long, 3.25 wide; opisthosoma 5.31 long, 3.49 wide. Eye sizes and interdistances: AME 0.32, ALE 0.25, PME 0.26, PLE, 0.27; AME–AME 0.18, AME–ALE 0.15, PME–PME 0.19, PME–PLE 0.39, ALE–PLE 0.07. MOA 0.80 long, anterior width 0.72, posterior width 0.68. Clypeus height 1.14. Leg measurements: I 12.91 (3.39, 3.98, 3.09, 2.45); II 11.88 (3.15, 3.60, 2.93, 2.20); III 11.76 (3.03, 3.45, 3.28, 1.92); IV 15.17 (3.61, 4.43, 4.48, 2.65). Leg formula: 4123. Carapace deep brown, opisthosoma black, other characters same as in male.

***Epigyne*** (Fig. 7). Median plate small, nearly trapezoidal, with nearly straight posterior margin; copulatory openings hidden in a groove. Spermathecae oval; copulatory ducts thick; slender fertilization ducts hidden in dorsal view.

##### Distribution.

Known only from the type locality, Xizang, China.

#### ﻿ Mallinella yadong sp. nov.

Taxon classificationAnimaliaAraneaeZodariidae

DC574CE8-D4E6-51BE-8ECB-DCC55A204903

https://zoobank.org/F4D1E17D-4B90-403A-80C6-47C5B0121A18

**Figure 8.**
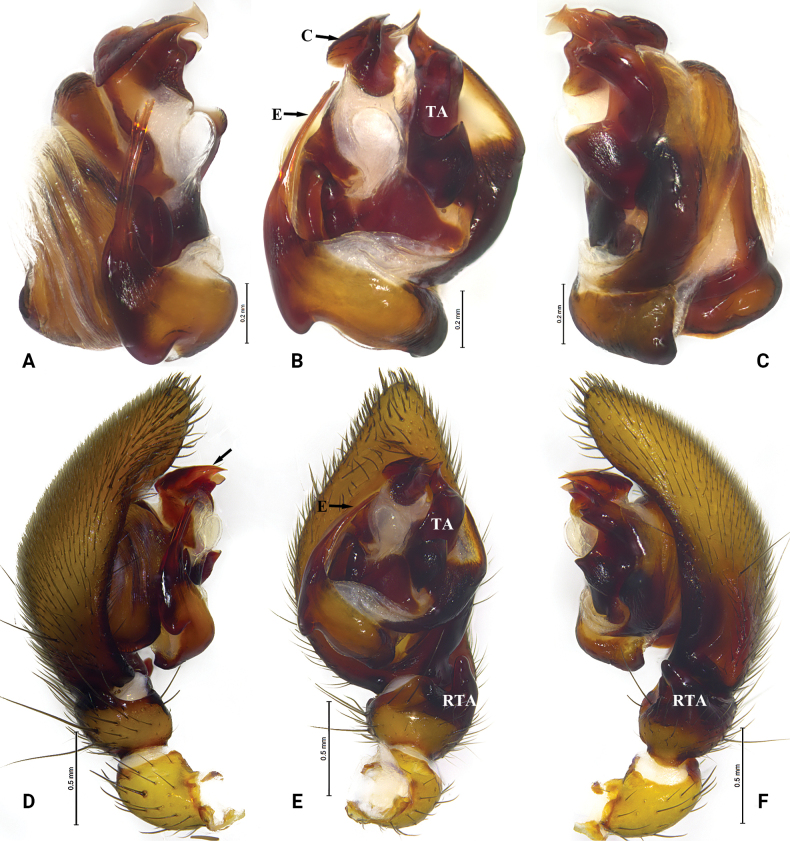
*Mallinellayadong* sp. nov. **A–C** paratype male **D–F** holotype male **A** male left bulb, prolateral view **B** same, ventral view **C** same, retrolateral view **D** male left palp, prolateral view **E** same, ventral view **F** same, retrolateral view. Abbreviations: C = conductor; E = embolus; RTA = retrolateral tibial apophysis; TA = tegular apophysis.

**Figure 9.**
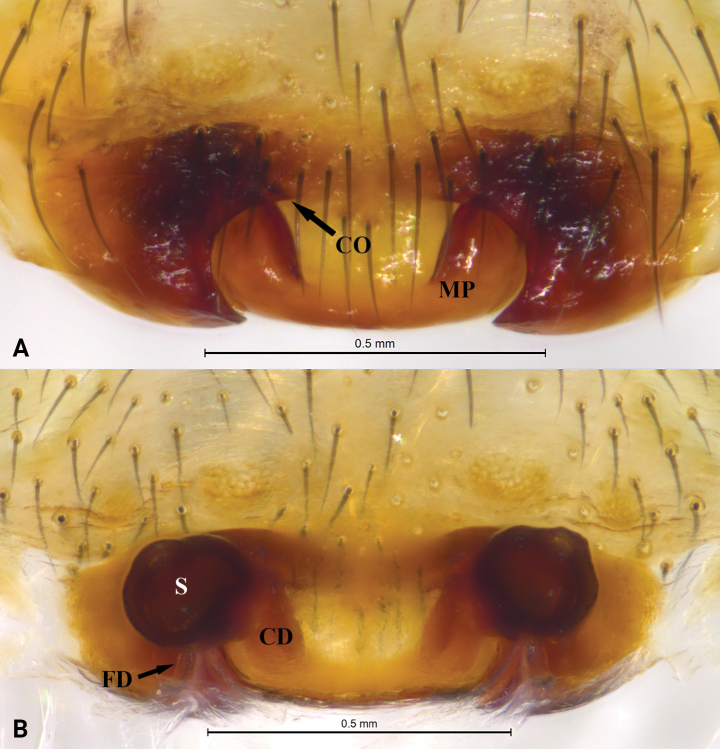
*Mallinellayadong* sp. nov., paratype female **A** epigyne, ventral view **B** same, dorsal view. Abbreviations: CD = copulatory duct; CO = copulatory opening; FD = fertilization duct; MP = median plate; S = spermatheca.

**Figure 10.**
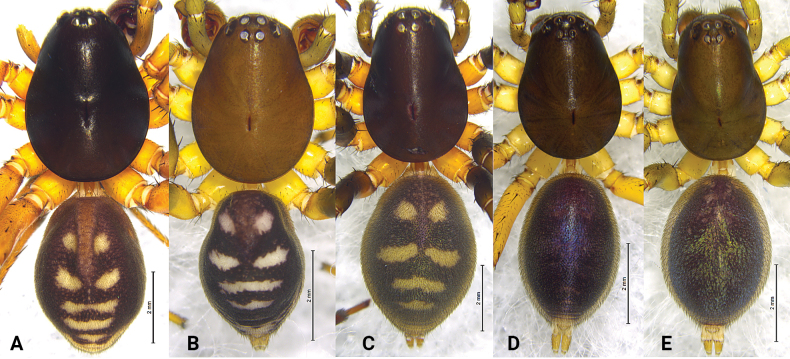
*Mallinella* spp. habitus, dorsal view **A***Mallinellamigu* sp. nov., male holotype **B***Mallinellamеdog* sp. nov., male holotype **C***Mallinellamеdog* sp. nov., female paratype **D***Mallinellayadong* sp. nov., male holotype **E***Mallinellayadong* sp. nov., female paratype.

Figs 8 , 9 , 10D, E Chinesse name: 亚东马利蛛

##### Type material.

***Holotype***: China • ♂; Xizang, Yadong County; 27°28′58″N, 88°54′14″E; elev. 3044 m; 10 July 2018; L. Wang, Z. Wu & Y. Mu leg.; SWUCT-ZOD-05-01.

***Paratypes***: China – Xizang • 1♂ 1♀; same data as holotype; SWUCT-ZOD-05-02 and SWUCT-ZOD-05-03 • 1♀; Xiayadong Township, Asang; 27°24′20″N, 88°57′10″E; elev. 2870 m; 11 July 2018; L. Wang, Z. Wu & Y. Mu leg.; SWUCT-ZOD-05-04.

##### Etymology.

The specific name is derived from the type locality; it is a noun in apposition.

##### Diagnosis.

The male of this new species resembles *Mallinellauncinata* (Ono, 1983) (Ono 1983: 214, figs 5–8), but it differs by the anterior part of the tegular apophysis projecting laterally and rotated (vs not projecting laterally and not rotated) and short embolus (vs long) (Fig. 8). The female of this new species resembles *M.laxa* Zhang & Zhang, 2019 (Zhang and Zhang 2019: 10, figs 8G, H, 9F, G), but differs from it by the thinner copulatory ducts (vs thick copulatory ducts) and median plate with a trapezoidal depression (vs V-shaped depression) (Fig. 9).

##### Description.

**Male** holotype (Fig. 10D) total length 7.80. Prosoma 3.66 long, 2.52 wide; Opisthosoma 3.94 long, 2.51 wide. Eye sizes and interdistances: AME 0.18, ALE 0.20, PME 0.20, PLE 0.23; AME–AME 0.14, AME–ALE 0.13, PME–PME 0.16, PME–PLE 0.27, ALE–PLE 0.08. MOA 0.64 long, anterior width 0.55, posterior width 0.53. Clypeus height 0.82. Leg measurements: I 11.56 (3.00, 3.52, 2.85, 2.19); II 10.75 (2.88, 3.22, 2.69, 1.96); III 10.45 (2.71, 2.94, 3.05, 1.75); IV 13.27 (3.10, 3.82, 4.11, 2.24). Leg formula: 4123. Carapace and fovea black, slightly swollen. Radial grooves distinct. Legs yellow. Opisthosoma oval, longer than wide, dorsum without any markings. Dorsal scutum indistinct. Spinnerets pale yellow.

***Palp*** (Fig. 8). Tibia with two apophyses, ventral tibial apophysis small, arc-shaped; retrolateral tibial apophysis digitiform, curved ventrally. Tegular apophysis thin, apico-prolateral process projecting laterally, rotated, baso-retrolateral process trapezoid in retrolateral view. Embolus short, bifurcate, lateral ramus shorter than mesal ramus. Conductor sclerotized, apex of conductor irregularly fluctuating.

**Female** (Fig. 10E) total length 7.96. Prosoma 3.82 long, 2.47 wide; opisthosoma 4.04 long, 2.77 wide. Eye sizes and interdistances: AME 0.18, ALE 0.19, PME 0.22, PLE, 0.18; AME–AME 0.12, AME–ALE 0.14, PME–PME 0.16, PME–PLE 0.28, ALE–PLE 0.11. MOA 0.59 long, anterior width 0.49, posterior width 0.56. Clypeus height 0.78. Leg measurements: I 8.98 (2.43, 2.92, 1.98, 1.65); II 8.62 (2.33, 2.71, 1.93, 1.65); III 8.55 (2.19, 2.68, 2.26, 1.42); IV 10.73 (2.66, 3.24, 3.09, 1.74). Leg formula: 4123. Other characters same as male except lighter color than male.

***Epigyne*** (Fig. 9). Median plate large, with trapezoidal depression and slightly cambered posterior margin; copulatory openings hidden in groove. Spermathecae oval; copulatory ducts thin; slender fertilization ducts visible, directed posteriorly.

##### Distribution.

Known only from the type locality, Xizang, China.

## Supplementary Material

XML Treatment for Asceua chayu

XML Treatment for Asceua dawai

XML Treatment for Cydrela linzhiensis

XML Treatment for Mallinella migu

XML Treatment for Mallinella mеdog

XML Treatment for Mallinella yadong

## ﻿Additional information

### Author ORCIDs

Lu-Yu Wang https://orcid.org/0000-0002-5250-3473

Yan-Nan Mu https://orcid.org/0000-0002-2504-673X

Feng Lu https://orcid.org/0000-0001-6291-9117

Yong-Qiang Xu https://orcid.org/0000-0002-1889-9531

Zhi-Sheng Zhang https://orcid.org/0000-0002-9304-1789

### Data availability

All of the data that support the findings of this study are available in the main text.

## References

[B1] Álvarez-Padilla F Hormiga G (2007) A protocol for digesting internal soft tissues and mounting spiders for scanning electron microscopy. The Journal of Arachnology 35(3): 538–542. 10.1636/Sh06-55.1

[B2] Hu JL (2001) Spiders in Qinghai-Tibet Plateau of China. Henan Science and Technology Publishing House, Henan, 658 pp.

[B3] Hu JL Li AH (1987a) The spiders collected from the fields and the forests of Xizang Autonomous Region, China. (1). In: Zhang S (Ed.) Agricultural Insects, Spiders, Plant Diseases and Weeds of Xizang.Vol. 1. The Tibet People’s Publishing House, Lhasa, 315–392.

[B4] Hu JL Li AH (1987b) The spiders collected from the fields and the forests of Xizang Autonomous Region, China. (II). In: Zhang S (Ed.) Agricultural Insects, Spiders, Plant Diseases and Weeds of Xizang.Vol. 2. The Tibet People’s Publishing House, Lhasa, 247–353.

[B5] Jin C Zhang F (2013) Two new *Mallinella* species from southern China (Araneae, Zodariidae). ZooKeys 296: 79–88. 10.3897/zookeys.296.4622 PMC3689113 23794879

[B6] Ono H (1983) Zodariidae aus dem Nepal-Himalaya. I. Neue Arten der Gattung *Storena* Walckenaer 1805 (Arachnida: Araneae). Senckenbergiana Biologica 63: 211–217.

[B7] Sankaran PM (2023) Taxonomic notes on the ant-eating spider genera *Asceua* Thorell, 1887 and *Cydrela* Thorell, 1873 from India, with comment on Indian species of *Euryeidon* Dankittipakul & Jocqué, 2004 (Araneae: Zodariidae). Zootaxa 5296(3): 381–405. 10.11646/zootaxa.5296.3.4 37518438

[B8] WSC (2024) World Spider Catalog. Version 25.0. Natural History Museum Bern. 10.24436/2 [Accessed 4 Feb. 2024]

[B9] Zhang BS Zhang F (2019) Four new species of the genus *Mallinella* (Araneae: Zodariidae) from Malaysia. Zootaxa 4568(2): 242–260. 10.11646/zootaxa.4568.2.2 31715856

